# Characterizing bacterial and fungal communities along the longitudinal axis of the intestine in cynomolgus monkeys

**DOI:** 10.1128/spectrum.01996-23

**Published:** 2023-11-08

**Authors:** Yunpeng Yang, Ning Xu, Linlin Yao, Yong Lu, Changshan Gao, Yanhong Nie, Qiang Sun

**Affiliations:** 1 College of Veterinary Medicine, Institute of Comparative Medicine, Yangzhou University, Yangzhou, China; 2 Jiangsu Co-Innovation Center for Prevention and Control of Important Animal Infectious Diseases and Zoonoses, Yangzhou University, Yangzhou, China; 3 CAS Key Laboratory of Primate Neurobiology, State Key Laboratory of Neuroscience, Institute of Neuroscience, CAS Center for Excellence in Brain Science and Intelligence Technology, Chinese Academy of Sciences, Shanghai, China; 4 Shanghai Center for Brain Science and Brain-Inspired Intelligence Technology, Shanghai, China; 5 University of Chinese Academy of Sciences, Beijing, China; China Agricultural University, Beijing, China

**Keywords:** gut microbiota, cynomolgus monkeys, gut biogeography, bacterial and fungal functions, correlations

## Abstract

The gut microbiota develops specialized local communities along the gastrointestinal (GI) tract of the host. Among these microbes, intestinal bacteria and fungi interact closely with the host and play vital roles in their health. However, the gut biogeography of bacteria and fungi has rarely been studied simultaneously, and their correlations have not been reported. In this study, we collected the intestinal luminal contents from different gastrointestinal regions (the ileum, caecum, and colon) of wild-type female cynomolgus monkeys and performed 16S and 18S rRNA gene sequencing to determine the gut biogeography of bacterial and fungal communities. The richness and diversity of bacteria and fungi increased gradually from the small to the large intestine. The bacterial and fungal composition of ileum differed significantly from that of the colon. Specifically, Bacteroidia and Spirochaetia were abundant in the caecum and colon, while Actinobacteria and Cyanobacteria were enriched in the ileum. As for fungal taxa, the genera *Aspergillus*, *Wallemia*, and *Cryptococcus_f_Tremellaceae* were enriched in the colon, while the genus *Fusarium* was abundant in the caecum. Consequently, the bacterial and fungal functions of the ileum differed from those of the caecum and colon. Notably, we characterized the correlations between intestinal bacteria and fungi at different gastrointestinal regions of cynomolgus monkeys using Spearman correlation analysis. In summary, our results contribute to a more comprehensive understanding of the composition and function of intestinal bacteria and fungi in non-human primates (NHPs) and reveal their correlations along the longitudinal axis of the intestine.

**IMPORTANCE:**

Gut microbiota varies along the gastrointestinal (GI) tract and exerts profound influences on the host’s physiology, immunity, and nutrition. Given that gut microbes interact with the host closely and the gastrointestinal function differed from the small to the large intestine, it is essential to characterize the gut biogeography of the microbial community. Here, we focused on intestinal bacteria and fungi in cynomolgus monkeys and determined their spatial distribution along the GI tract by performing 16S and 18S rRNA gene sequencing. The composition and function of bacterial and fungal communities differed significantly at different biogeographic sites of the intestine, and the site-specific correlations between intestinal bacteria and fungi were revealed. Thus, our studies characterized the gut biogeography of bacteria and fungi in NHPs and revealed their site-specific correlations along the GI tract.

## INTRODUCTION

The gut microbiota represents a dynamic ecosystem containing bacteria, viruses, fungi, yeasts, and other single-celled organisms (i.e., archaea and protozoans). The microbiota interacts with the host closely and is essential for sustaining normal physiology and health (1, 2). As reported, the gut microbial composition may be impacted by various factors, such as sex (3), diet (4, 5), aging (6 – 8), seasonal factor (9), delivery mode (natural birth or cesarean section) (10), and host genetics (11, 12). It has also been reported that the gut microbial composition is variable along the longitudinal axis of the intestine (13). Since the gastrointestinal function of the host varies from the small to the large intestine and the expression of the host gene can be regulated by gut microbiota along the length of the gut, it is essential to characterize the composition and function of site-specific microbial communities along the GI tract (14).

To date, the specialized bacterial communities in the intestine have been studied in various species. In human beings, the bacterial biogeography throughout the broad sites (mouth, stomach/duodenum, colon, and stool) of the gastrointestinal tract was studied in two men and two women (15). The compositional and functional differences of mucosal microbiota along the intestine were studied in five healthy Swedish adults (16). The biogeography of gut microbiome and its impact on geographically variable immune responses was studied in 2-year-old children from across different cohorts, highlighting population-specific interventions for the improvement of child health (17). In newborn piglets, distinct microbial communities were discovered through the analysis of the temporal and spatial dynamics of the gut microbiome from across six intestinal segments (duodenum, jejunum, ileum, caecum, colon, and rectum) (18). In addition, to explain the effects of gut microbiota on adipogenesis and animal growth, the differences in the microbial structure were compared along the intestinal tract between two breeds of pigs (Landrace and Jinhua) (19).

A growing body of research suggests that a balanced fungal community is also required to sustain the health of the host (20 – 22). For example, dysbiosis of fungal microbiota can lead to various diseases, including alcoholic liver disease (23), allergic airway disease (24), and gut colitis (25 – 27). Therefore, it is important to characterize the local fungal communities along the GI tract.

Non-human primates (NHPs) share common genetic, physiological, and behavioral traits with human beings and are regarded as excellent models for scientific research and clinical application. While the gut bacteria composition of NHPs has been extensively investigated, only a handful of studies have focused on the biogeography of gut bacteria in NHPs. In rhesus macaques, although the mucosal and luminal bacteria from the jejunum, ileum, and ascending, transverse, and descending colons were studied, the bacterial microbes of the caecum were not investigated (28). In cynomolgus monkeys, the gut bacteria from different sites of the large intestine (caecum and ascending, transverse, and descending colons) had been studied; however, the bacterial community of the small intestine was uncharacterized (29). As for the fungal microbiome, a region-by-region taxonomic survey of intestinal fungi revealed that the *Kazachstania* genus and *K. pintolopesii* species dominated in both the small and large intestines of cynomolgus monkeys (30). While the composition of intestinal bacteria and fungi was studied along the GI tract of cynomolgus monkeys, the gut biogeography of bacteria and fungi has rarely been studied simultaneously, and their site-specific interactions have not been reported.

In this work, we performed 16S and 18S rRNA gene sequencing to determine the biogeography of bacterial and fungal communities along the longitudinal axis of the intestine in cynomolgus monkeys. Both the bacterial and fungal richness and diversity of luminal samples increased gradually from the ileum to the colon. Additionally, the bacterial and fungal composition of the ileum differed significantly from that of the colon. Bacteroidia and Spirochaetia were abundant in the caecum and colon, while Actinobacteria and Cyanobacteria were enriched in the ileum. As for fungal taxa, the genera *Aspergillus*, *Wallemia*, and *Cryptococcus_f_Tremellaceae* were enriched in the colon, while the genus *Fusarium* was abundant in the caecum. In accordance with the gut microbial changes, we uncovered that the functional roles of the bacterial and fungal communities in the ileum were different from those in the caecum and colon. Of note, the site-specific correlation analysis between intestinal bacteria and fungi revealed a close bacterial–fungal interaction in cynomolgus monkeys. Ultimately, our study characterized the gut biogeography of bacteria and fungi in cynomolgus monkeys and revealed their site-specific correlations along the length of the GI tract.

## RESULTS

### The richness and diversity of bacterial community along the GI tract

**Fig 1:**
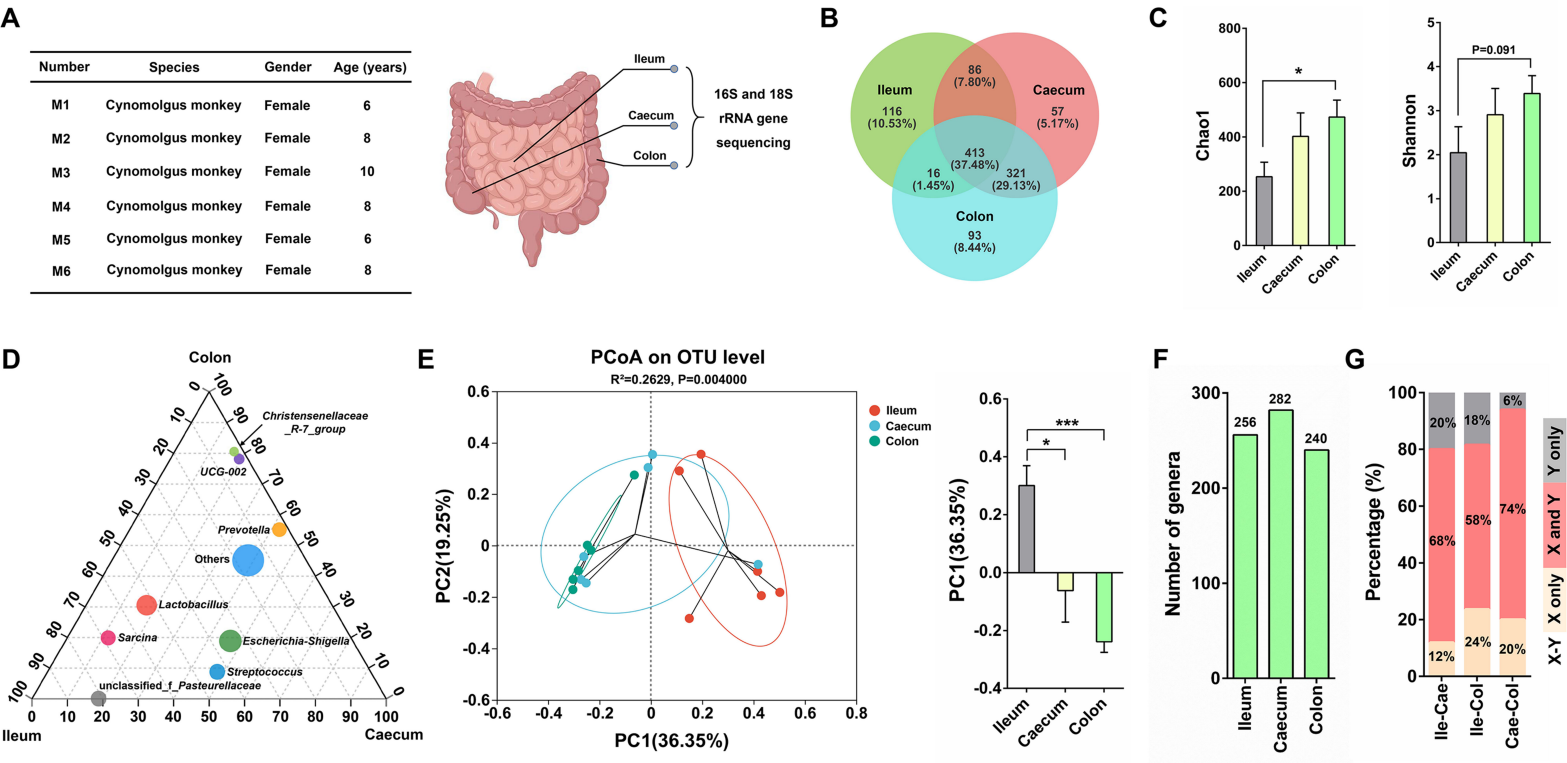
Richness and diversity of bacterial community along the gastrointestinal tract of cynomolgus macaques. (**A**) The basic information of monkeys used in this study and the diagram showing the sampling sites. (**B**) Venn diagram illustrating different OTUs between ileum, caecum, and colon. (**C**) The microbial richness (Chao1) and diversity (Shannon) of the ileum, caecum, and colon. (**D**) The ternary analysis based on the genus level revealed distinct bacterial compositions in the ileum, caecum, and colon samples. (**E**) Principal coordinate analysis of unweighted UniFrac distances between ileum, caecum, and colon. PC1 axis was analyzed to test dissimilarity between different groups. (**F**) The number of bacterial genera in ileum, caecum, and colon. (**G**) Percentage of shared bacterial genera (in pink) between sampling sites. Ile, ileum; Cae, caecum; Col, colon. X-Y represented Ile-Cae/Ile-Col/Cae-Col. By comparing the bacterial genera between two intestinal sites, the yellow bar (X only) indicated the proportion of bacterial genera that exist at one intestinal site (ileum or caecum), the gray bar (Y only) indicated the proportion of bacterial genera that exist at the other intestinal site (caecum or colon), the red bar (X and Y) indicated the proportion of bacterial genera that existed at both the two intestinal sites. Data are presented as mean ± SEM. The statistical significance of the data between two groups was analyzed using the *t*-test. *P < 0.05; ***P < 0.001.

The site-specific gut microbial profiles of cynomolgus monkeys were characterized by collecting luminal samples from three different gastrointestinal regions (ileum, caecum, and colon) of six wild-type female cynomolgus monkeys, which were used for 16S rRNA V3–V4 region amplicon sequencing (Fig. 1A). Venn diagram analysis showed that the number of operational taxonomic units (OTUs) in the caecum (877) and colon (843) was significantly higher than that in the ileum (631) (Fig. 1B). Of the total OTUs, 413 were shared by the ileum, caecum, and colon, while 116, 57, and 93 OTUs were exclusively found in the ileum, caecum, and colon, respectively (Fig. 1B). Furthermore, the bacterial richness (Chao1) and community diversity (Shannon) of the colon were higher than those of the ileum (Fig. 1C). The ternary analysis based on the genus level revealed distinct bacterial compositions in the ileum, caecum, and colon samples (Fig. 1D). The principal coordinate analysis (PCoA) of the unweighted UniFrac distances indicated significant differences in bacterial composition between the ileum and caecum/colon, with the principal component 1 (PC1) axis explaining a substantial proportion of the variability (Fig. 1E). Additionally, the caecum contained more bacterial genera than the ileum and colon (Fig. 1F). Then, the percentage of bacterial genera shared by the ileum and caecum (Ile-Cae), ileum and colon (Ile-Col), and caecum and colon (Cae-Col) was studied, respectively (Fig. 1G). The highest percentage of shared bacterial genera between two sampling sites was observed in Cae-Col (74%), followed by Ile-Cae (68%), and Ile-Col (58%) (Fig. 1G). These results suggest that the gut bacteria of the caecum may act as the transition stage from the ileum to the colon.

### Characterizing bacterial composition along the GI tract

**Fig 2:**
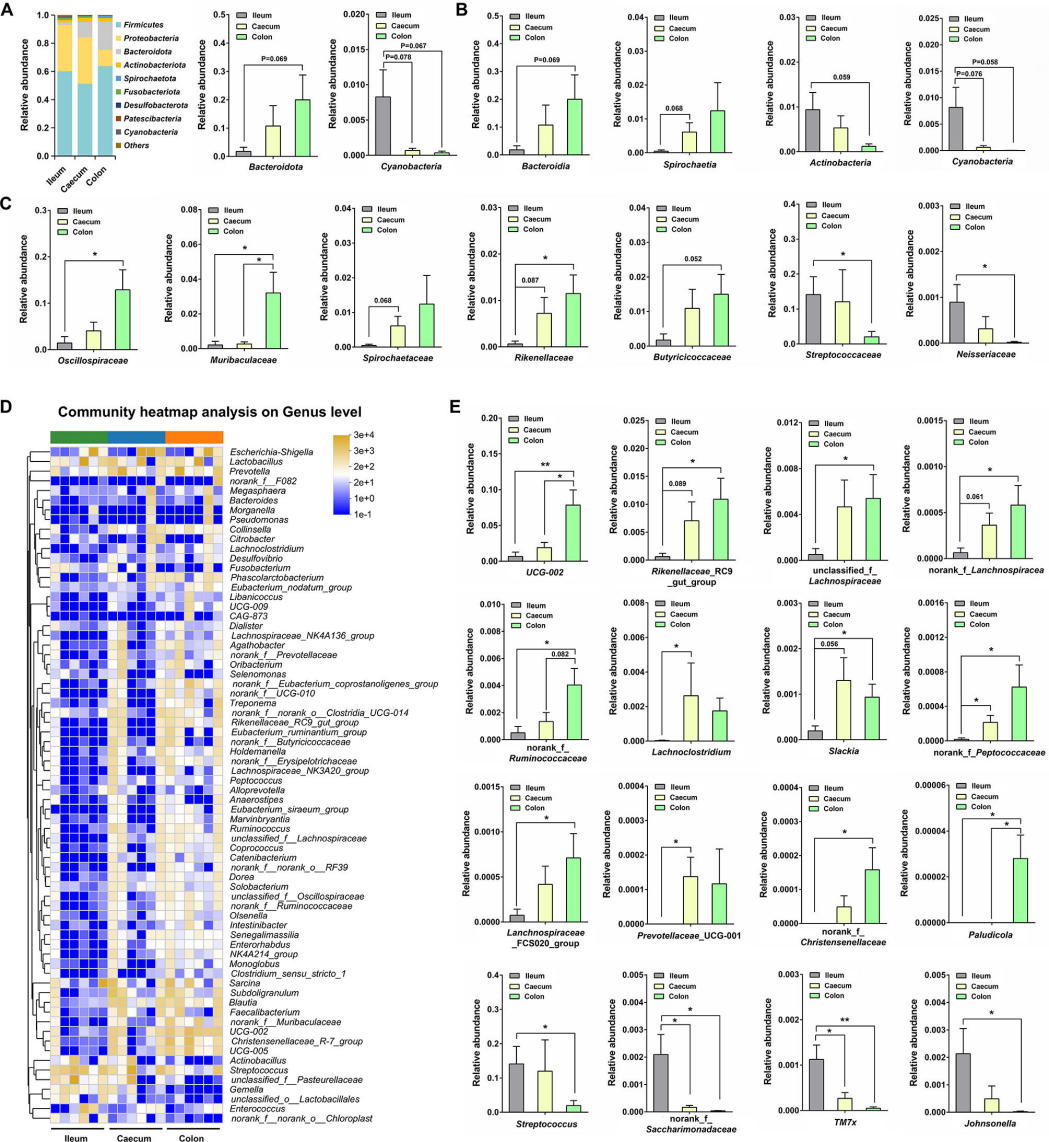
Comparison of bacterial composition between ileum, caecum, and colon. (**A**) Relative abundance of OTUs assigned at the phylum level. (**B**) Relative abundance of OTUs assigned at the class level. (**C**) Relative abundance of OTUs assigned at the family level. (**D**) Community heatmap analysis of top 70 genera between ileum, caecum, and colon. (**E**) Relative abundances of OTUs assigned at the genus level. Data are presented as mean ± SEM. The statistical significance of the data between two groups was analyzed using the *t*-test. *P < 0.05; **P < 0.01.

The differences in bacterial taxa between the ileum, caecum, and colon were examined at the phylum, class, family, and genus levels. At the phylum level, the relative abundance of Bacteroidota was higher in the caecum and colon compared to the ileum, while Cyanobacteria was enriched in the ileum (Fig. 2A). At the class level, Bacteroidia and Spirochaetia were abundant in the caecum and colon, while Actinobacteria and Cyanobacteria were enriched in the ileum (Fig. 2B). At the family level, Oscillospiraceae, Muribaculaceae, Spirochaetaceae, Rikenellaceae, and Butyricicoccaceae were abundant in the colon, whereas Streptococcaceae and Neisseriaceae were enriched in the ileum (Fig. 2C). The top 70 genera were selected and used to perform the heatmap analysis in order to examine the bacterial differences between the ileum, caecum, and colon at the genus level (Fig. 2D). Among these genera, *UCG-002*, *Rikenellaceae*_RC9_gut_group, unclassified_f_*Lachnospiraceae*, norank_f_*Lanchnospiracea*, norank_f_*Ruminococcaceae*, *Lachnoclostridium*, *Slackia*, norank_f_*Peptococcaceae*, *Lanchnospiraceae*_FCS020_group, *Prevotellaceae*_UCG-001, and norank_f_*Christensenellaceae* were enriched in the caecum and colon, while *Streptococcus*, norank_f_*Saccharimonadaceae*, *TM7x*, and *Johnsonella* were abundant in the ileum (Fig. 2E). Notably, *Paludicola* was only detected in the colon (Fig. 2E).

Linear discriminant analysis (LDA) effect size (LEfSe) was performed to identify the distinguishing bacterial taxa between the ileum, caecum, and colon (LDA score >4.0, *P* < 0.05). Pasteurellaceae, Staphylococcales, *Gemella*, and unclassified_g_*Streptococcus* were enriched in the ileum; Ruminococcaceae, *Blautia*, and Erysipelotrichales were abundant in the caecum; Bacteroidales, Oscillospiraceae, *UCG-002*, *UCG-005*, and Muribaculaceae were enriched in the colon (Fig. S1A). Moreover, a genus-based Spearman correlation matrix was generated to uncover bacterial interactions across different sampling sites. Compared with the bacteria in the ileum, most of the bacteria in the caecum and colon were positively correlated with each other (Fig. S1B, C and D).

### Characterizing bacterial function along the GI tract

**Fig 3:**
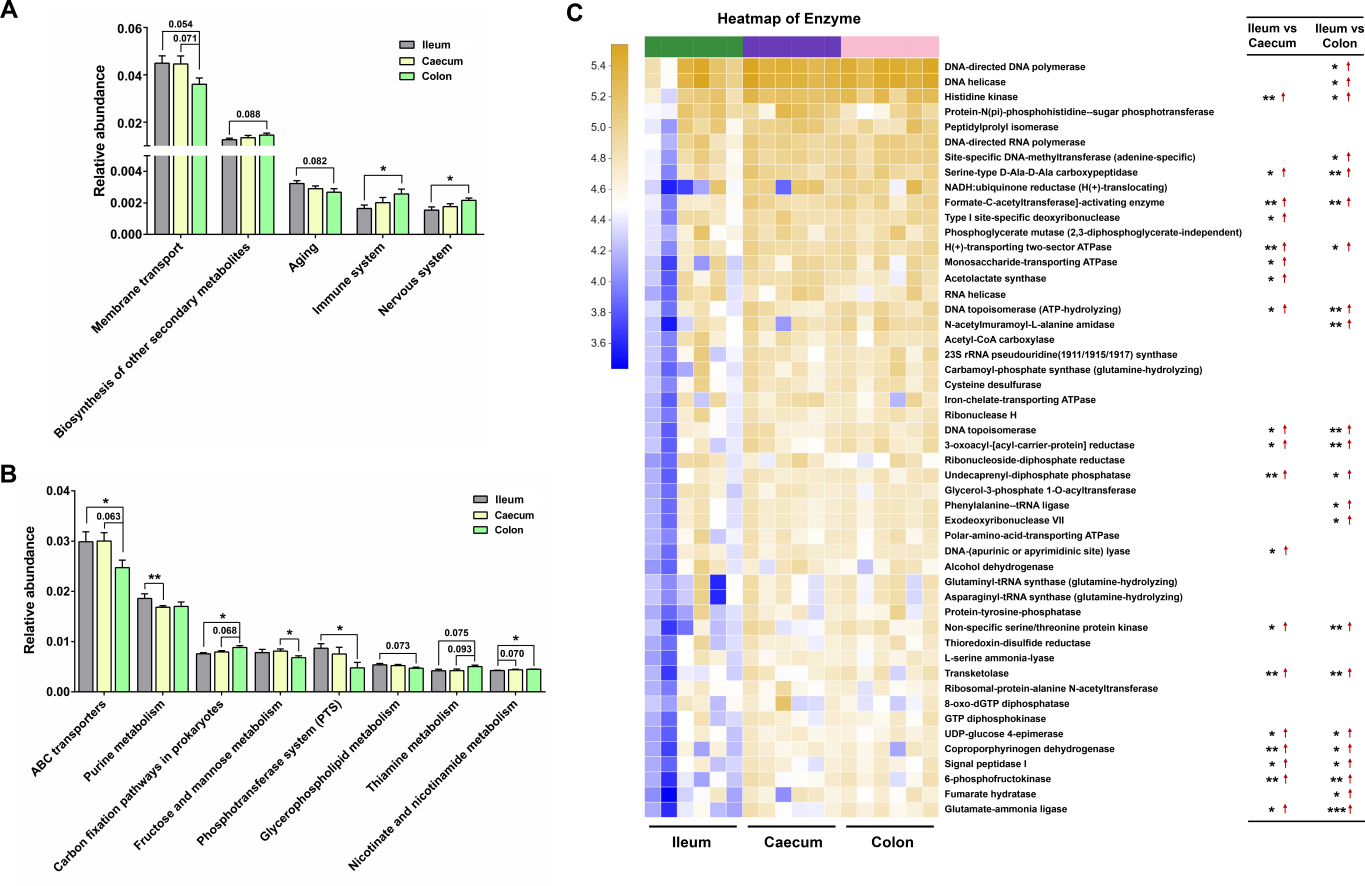
Functional differences of intestinal bacteria between the ileum, caecum, and colon. (**A** and **B**), PICRUSt2-predicted second- and third-level KEGG pathway abundances in the gut microbiota of the ileum, caecum, and colon. (**C**) Heatmap illustrating PICRUSt2-predicted microbial enzyme differences between the ileum, caecum, and colon. The statistical significance between the ileum and caecum/colon was listed at the right. Red arrows (↑) represented improved microbial enzymes in the caecum or colon. Data are presented as the mean ± SEM. The statistical significance of the data between two groups was analyzed using the *t*-test, *P < 0.05; **P < 0.01; ***P < 0.001.

The function of bacterial communities was analyzed using Phylogenetic Investigation of Communities by Reconstruction of Unobserved States (PICRUSt2). Across the second-level Kyoto Encyclopedia of Genes and Genomes (KEGG) pathways, the functions related to membrane transport and aging were enriched in the ileum, while the functions related to the biosynthesis of other secondary metabolites, immune system, and nervous system were abundant in the colon (Fig. 3A). Among the third-level KEGG pathways, the functions related to ABC transporters, purine metabolism, fructose and mannose metabolism, and phosphotransferase system (PTS) were enriched in the ileum, while carbon fixation pathways in prokaryotes, thiamine metabolism, and nicotinate and nicotinamide metabolism were abundant in the colon (Fig. 3B). In focusing on the top 50 PICRUSt2-predicted bacterial enzymes, it was found that many of these enzymes were not abundant in the ileum (Fig. 3C). These findings indicated that the functions of ileal bacteria were different from the colonic bacteria.

### The richness and diversity of fungal community along the GI tract

**Fig 4:**
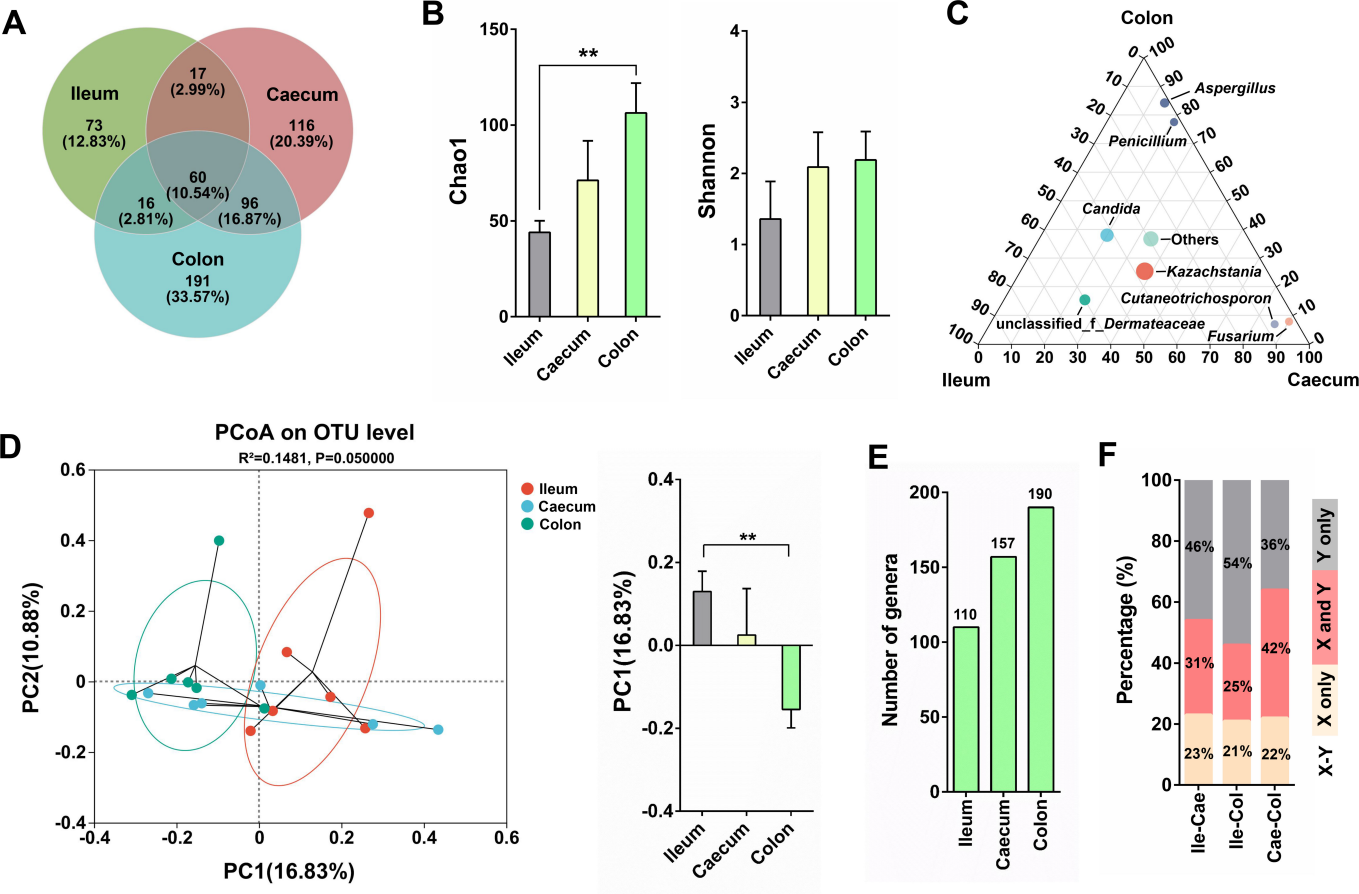
Richness and diversity of the fungal community along the gastrointestinal tract of cynomolgus macaques. (**A**) Venn diagram for different OTUs between the ileum, caecum, and colon. (**B**) The richness and sample diversity in ileum, caecum, and colon. (**C**) The ternary analysis based on the genus level revealed distinct fungal compositions in the ileum, caecum, and colon samples. (**D**) Principal coordinate analysis of unweighted UniFrac distances between ileum, caecum, and colon. PC1 axis was analyzed to test dissimilarity between different groups. (**E**) The number of fungal genera in ileum, caecum, and colon. (**F**) Percentage of shared fungal genera (in pink) between different sampling sites. Ile, ileum; Cae, caecum; Col, colon. X-Y represented Ile-Cae/Ile-Col/Cae-Col. By comparing the fungal genera between two intestinal sites, the yellow bar (X only) indicated the proportion of fungal genera that exist at one intestinal site (ileum or caecum), the gray bar (Y only) indicated the proportion of fungal genera that exist at the other intestinal site (caecum or colon), and the red bar (X and Y) indicated the proportion of fungal genera that existed at both the two intestinal sites. Data are presented as the mean ± SEM. The statistical significance of the data between two groups was analyzed using the *t*-test. **P < 0.01.

To characterize the fungal community along the GI tract of cynomolgus monkeys, the ITS2 region of fungal 18S rDNA was amplified and sequenced using an Illumina MiSeq platform (Fig. 1A). Venn diagram analysis indicated that the number of OTUs in the caecum (289) and colon (363) was significantly higher than those in the ileum (166), and 73, 116, and 191 OTUs exclusively belonged to the ileum, caecum, and colon, respectively (Fig. 4A). The richness (Chao1) and diversity (Shannon) of fungal microbes were higher in the colon than the ileum (Fig. 4B). The ternary analysis based on the genus level revealed distinct fungal composition in the ileum, caecum, and colon (Fig. 4C). Principal coordinate analysis (PCoA) of the unweighted UniFrac distances indicated significant differences in fungal composition between the ileum and colon, with the PC1 axis explaining a substantial proportion of the variability (Fig. 4D). Additionally, as shown in Fig. 4E, the number of fungal genera increased gradually from the ileum to colon, indicating that the fungal diversity was generally higher in the large intestine. Similar to gut bacteria, the highest percentage of shared fungal genera between any two sampling sites was observed in Cae-Col (42%), followed by Ile-Cae (31%), and Ile-Col (25%) (Fig. 4F).

### Characterizing fungal composition and function along the GI tract

**Fig 5:**
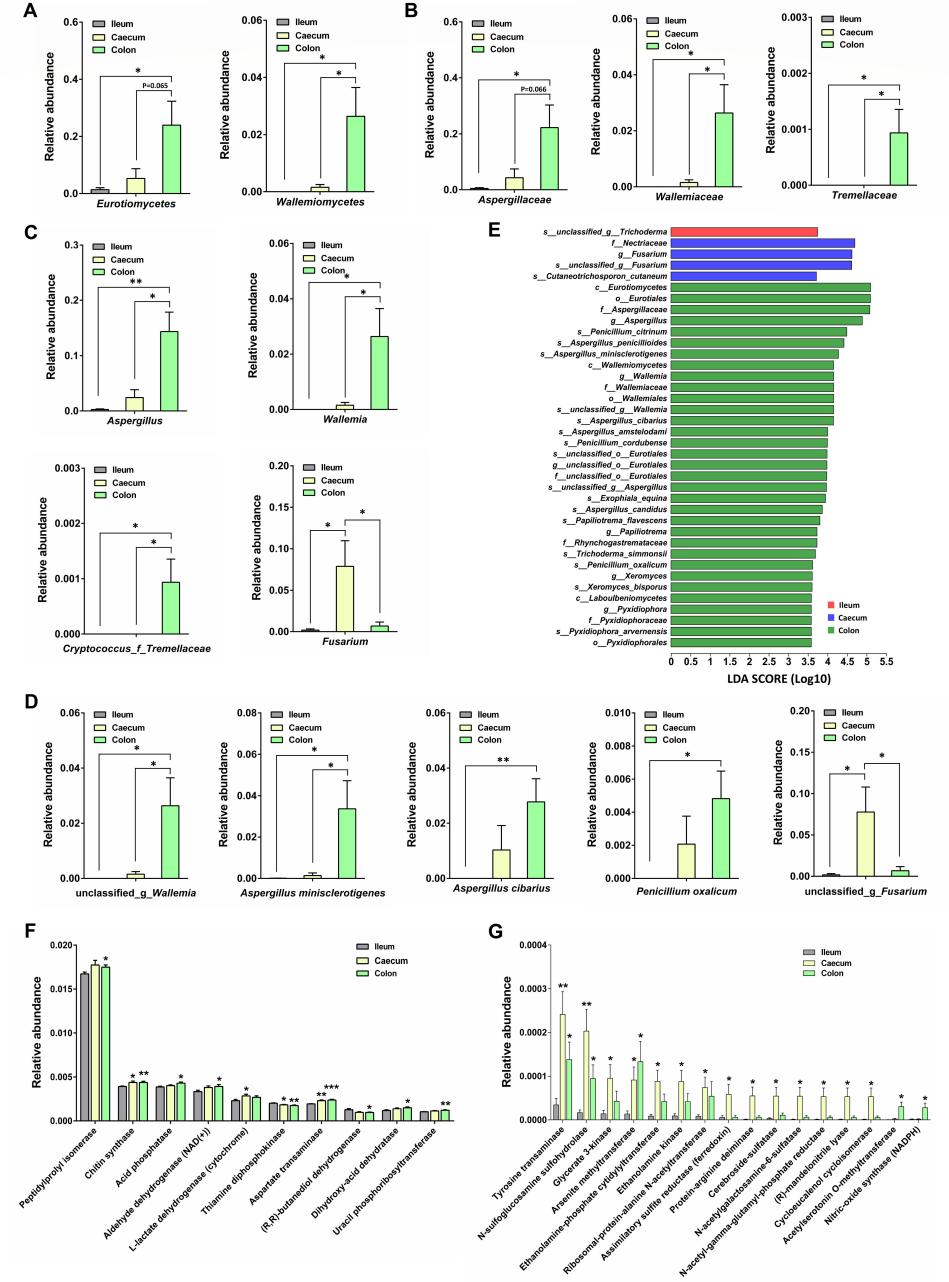
Comparing the composition and function of the fungal community between the ileum, caecum, and colon. Relative abundance of OTUs was assigned at the class (**A**), family (**B**), genus (**C**), and species (**D**) levels. (**E**) LEfSe analysis to characterize the taxonomic differences of fungal microbes between the ileum, caecum, and colon. LDA score cut-off was set as 3.5 (*P* < 0.05). (**F**) PICRUSt2-predicted top 10 differential enzymes between the ileum and caecum/colon. (**G**) PICRUSt2-predicted differential enzymes with more than fivefold change between the ileum and caecum/colon. Data are presented as the mean ± SEM. The statistical significance of the data between two groups was analyzed using the *t*-test. *P < 0.05; **P < 0.01; ***P < 0.001.

The differences in fungal taxa between the ileum, caecum, and colon were examined at the class, family, genus, and species levels. At the class level, Eurotiomycetes and Wallemiomycetes were enriched in the colon (Fig. 5A). At the family level, Aspergillaceae, Wallemiaceae, and Tremellaceae were abundant in the colon (Fig. 5B). Accordingly, the genera *Aspergillus*, *Wallemia*, and *Cryptococcus_f_Tremellaceae* were enriched in the colon, while the genus *Fusarium* was abundant in the caecum (Fig. 5C). At the species level, unclassified_g_*Wallemia*, *Aspergillus minisclerotigenes*, *Aspergillus cibarius*, and *Penicillium oxalicum* were enriched in the colon, while unclassified_g_*Fusarium* was abundant in the caecum (Fig. 5D). LEfSe was performed to identify the distinguishing fungal taxa between the ileum, caecum, and colon (LDA score >3.5, *P* < 0.05). As shown in Fig. 5E, unclassified_g_*Trichoderma* was enriched in the ileum; Nectriaceae, *Fusarium*, and *Cutaneotrichosporon cutaneum* were abundant in the caecum; *Aspergillus*, *Wallemia*, *Papiliotrema*, *Aspergillus cibarius*, *Aspergillus amstelodami*, *Penicillium cordubense*, and *Exophiala equina* were enriched in the colon. A genus-based Spearman correlation analysis indicated that most of the fungi in the ileum and caecum were positively correlated with each other (Fig. S2).

PICRUSt2 was used for functional analysis of fungal communities in the ileum, caecum, and colon. In focusing on the top 10 differential fungal enzymes, we found that peptidylprolyl isomerase, chitin synthase, acid phosphatase, aldehyde dehydrogenase (NAD(+)), L-lactate dehydrogenase (cytochrome), aspartate transaminase, dihydroxy-acid dehydratase, and uracil phosphoribosyltransferase were enriched in the colon, while thiamine diphosphokinase and (R,R)-butanediol dehydrogenase were abundant in the ileum (Fig. 5F). Additionally, the enzymes with more than fivefold change between the ileum and caecum/colon were analyzed (Fig. 5G). Among these enzymes, tyrosine transaminase, N-sulfoglucosamine sulfohydrolase, glycerate 3-kinase, arsenite methyltransferase, ethanolamine-phosphate cytidylyltransferase, ethanolamine kinase, and ribosomal-protein-alanine N-acetyltransferase were enriched in the caecum and colon; assimilatory sulfite reductase (ferredoxin), protein-arginine deiminase, cerebroside-sulfatase, N-acetylgalactosamine-6-sulfatase, N-acetyl-gamma-glutamyl-phosphate reductase, (R)-mandelonitrile lyase, and cycloeucalenol cycloisomerase were abundant in only the caecum; acetylserotonin O-methyltransferase and nitric-oxide synthase (NADPH) were exclusively enriched in the colon (Fig. 5G).

### Characterizing the correlations between bacterial and fungal genera along the GI tract

**Fig 6:**
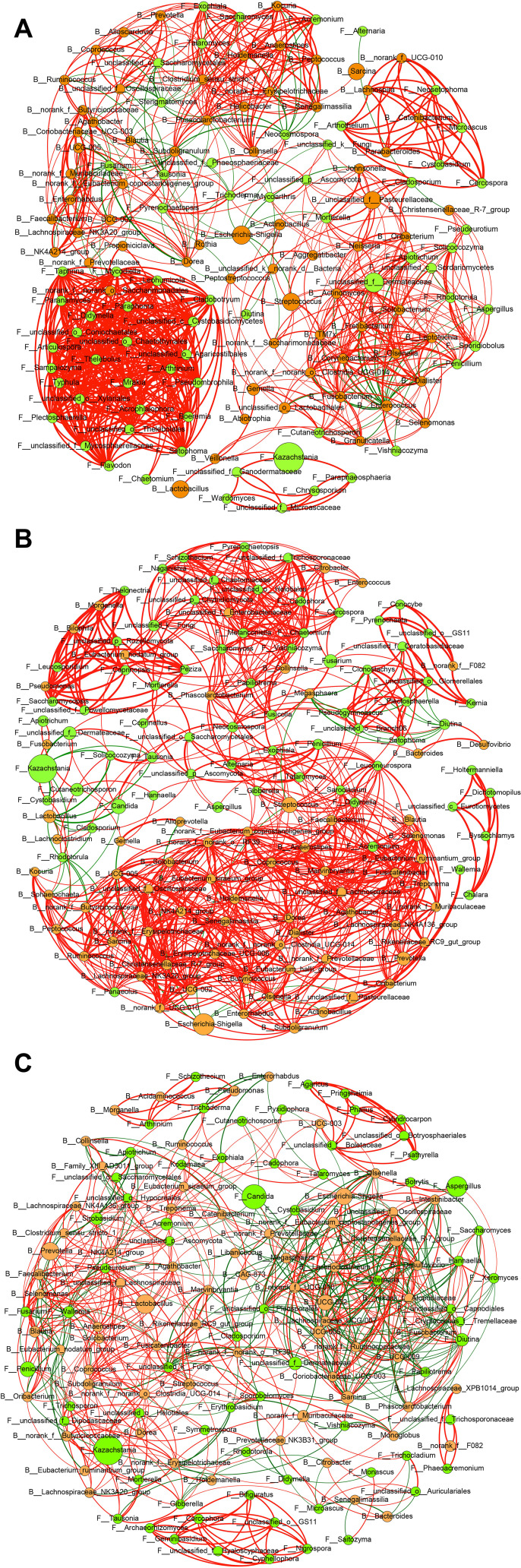
Correlation network of bacterial and fungal genera in the ileum (**A**), caecum (**B**), and colon (**C**). The correlation network was performed by using Gephi software (version 0.9.4). A connection is indicated for Spearman’s correlation with a coefficient >0.6 (positive correlation) or <−0.6 (negative correlation) and a significant (*P* < 0.05) correlation. Bacteria are labeled as a yellow node; fungi are labeled as a green node. The size of each node is proportional to the relative abundance. The red lines represent positive correlations between the nodes, and the green lines represent negative correlations, with the line width indicating the correlation magnitude.

We performed Spearman correlation analysis to uncover the bacterial–fungal correlations throughout the GI tract of cynomolgus monkeys (Tables S1 to S3). In the ileum, while there were significant positive correlations between different fungal genera, close correlations between bacterial and fungal genera were also observed (Fig. 6A). The bacterial genus unclassified_f*_Pasteurellaceae* was positively correlated with the fungal genera *Aggregatibacter*, *Johnsonella*, *Neisseria*, *Oribacterium*, *Apiotrichum*, *Cladosporium*, *Mortierella*, unclassified_f*_Dermateaceae*, unclassified_k*_Fungi*, and unclassified_p*_Ascomycota. Escherichia–Shigella* was negatively correlated with the fungal genera *Pyrenochaetopsis*, *Tausonia*, *Trichoderma*, and unclassified_k*_Fungi. Streptococcus* was positively correlated with the fungal genus *Mortierella*. As for fungal genera, *Kazachstania* was positively correlated with the bacterial genus *Lactobacillus*. unclassified_f*_Dermateaceae* was positively correlated with the bacterial genera *Actinomyces*, *Aggregatibacter*, *Corynebacterium*, *Dialister*, *Fretibacterium*, *Fusobacterium*, *Leptotrichia*, *Neisseria*, norank_f_norank_o*_Clostridia_UCG-014*, *Olsenella*, *Oribacterium*, *Solobacterium*, *TM7x*, and unclassified_f_*Pasteurellaceae*, while negatively correlated with *Enterococcus*. Unclassified_o_*Saccharomycetales* was positively correlated with the bacterial genera *Agathobacter*, *Alloscardovia*, *Blautia*, *Clostridium_sensu_stricto_1*, *Coprococcus*, *Helicobacter*, *Phascolarctobacterium*, *Prevotella*, *Ruminococcus*, and unclassified_f_*Oscillospiraceae*. Notably, *Arthothelium*, *Neosetophoma*, *Microascus*, *Cystobasidium*, and *Cercospora* interacted with the bacterial genera *Lachnospira*, norank_f*_UCG-010*, *Catenibacterium*, and *Parabacteroides* closely and established a positively correlated network.

In the caecum, a positively correlated network between different bacterial genera was observed (Fig. 6B). With respect to bacterial–fungal interactions, the bacterial genus *Streptococcus* was positively correlated with the fungal genera *Hannaell*a, unclassified_o*_Branch06*, *Clonostachys*, *Pseudogymnoascus*, *Setophoma*, and *Plectosphaerella. Lactobacillus* was positively correlated with the fungal genus *Kazachstania* and negatively correlated with *Candida*, *Solicoccozyma*, *Rhodotorula*, *Tausonia*, and *Cladosporium. Enterococcus* was positively correlated with the fungal genera *Cercospora*, *Chaetomium*, *Cadophora*, *Melanconiella*, unclassified_o*_Helotiales*, and unclassified_f*_Trichosporonaceae. Citrobacter* was positively correlated with the fungal genera *Cercospora*, *Cadophora*, *Schizothecium*, *Fusarium*, *Chaetomium*, *Melanconiella*, unclassified_o*_Helotiales*, unclassified_f_*Trichosporonaceae*, unclassified_f*_Chaetomiaceae*, and *Pyrenochaetopsis*. Additionally, *Phascolarctobacterium* was positively correlated with the fungal genera unclassified_k*_Fungi*, *Chaetomium*, *Collinsella*, *Alternaria*, *Exophiala*, *Fusicolla*, *Papiliotrema*, *Vishniacozyma*, *Saccharomyces*, unclassified_p*_Chytridiomycota*, *Naganishia*, *Melanconiella*, unclassified_o*_Helotiales*, unclassified_f*_Trichosporonaceae*, and *Cadophora*. Of note, a positively correlated network was established between specific bacteria (*Pseudomonas*, *Bilophila*, *Morganella*, and *Eubacterium_nodatum_group*) and fungi (*Saccharomycopsis*, unclassified_f*_Powellomycetaceae*, *Leucosporidium*, *Thelonectria*, unclassified_p*_Rozellomycota*, *Coprinopsis*, and *Peziza*) at the genus level (Fig. 6B).

In the colon, the bacterial genus *Lactobacillus* was positively correlated with the fungal genera *Penicillium*, *Symmetrospora*, *Trichosporon*, unclassified_f*_Dipodascaceae*, unclassified_p*_Ascomycota*, *Acremonium*, and unclassified_o*_Hypocreales. Prevotella* was positively correlated with the fungal genera *Acremonium*, *Sirobasidium*, *Fusarium*, *Exophiala*, unclassified_p*_Ascomycota*, and unclassified_o*_Hypocreales. UCG-002* was negatively correlated with the fungal genera *Hannaella*, *Cryptococcus_*f*_Tremellaceae*, and *Alternaria. Escherichia–Shigella* was positively correlated with unclassified_f*_Dermateaceae*, *Cystobasidium*, *Papiliotrema*, unclassified_o*_Pleosporales*, and *Alternaria* while negatively correlated with *Pyxidiophora*. With respect to the fungal genera, *Kazachstania* was positively correlated with bacterial genera *Eubacterium_ruminantium_group*, *Fusicatenibacter*, *Lachnospiraceae_NK3A20_group*, norank_f*_Butyricicoccaceae*, norank_f*_Erysipelotrichaceae*, norank_f_norank_o*_Clostridia_UCG-014*, norank_f_norank_o_*RF39*, and *Subdoligranulum*, while negatively correlated with *Eubacterium_nodatum_group. Candida* was negatively correlated with the bacterial genus norank_f*_Muribaculaceae; Aspergillus* was positively correlated with the bacterial genus *UCG-003. Penicillium* was positively correlated with the bacterial genus *Lactobacillus* and negatively correlated with *Clostridium_sensu_stricto_1* (Fig. 6C)

## DISCUSSION

Currently, most gut microbiome studies focus on analyzing fecal samples for microbiota screening. However, since the gut microbiota forms specialized local communities along the GI tract and interacts closely with the host, it is important to characterize site-specific microbial communities along the longitudinal axis of the intestine. In this study, we investigated the gut biogeography of bacteria and fungi and uncovered their correlations along the GI tract of cynomolgus monkeys.

To characterize the variation of bacterial and fungal communities along the longitudinal axis of the gut in cynomolgus monkeys, the top six bacterial and fungal genera in the ileum, caecum, and colon were ranked according to their relative abundance and listed in Fig. S3A. For luminal bacterial genera, *Escherichia–Shigella* and *Lactobacillus* were abundant in the ileum, caecum, and colon; *Streptococcus* and unclassified_f_Pasteurellaceae were enriched in the ileum and caecum; *Prevotella* was abundant in the caecum and colon. As to *UCG-002,* it was mainly identified in the colon. For luminal fungal genera, *Kazachstania*, *Candida*, and unclassified_f_Dermateaceae were abundant in the ileum, caecum, and colon; *Apiotrichum*, *Mortierella*, and *Cladosporium* were abundant in the ileum; *Fusarium*, *Cutaneotrichosporon*, and unclassified_k_Fungi were abundant in the caecum; *Aspergillus*, *Penicillium*, and *Acremonium* were enriched in the colon. These results further emphasized the importance of analyzing the biogeography of bacterial and fungal communities of cynomolgus monkeys.

When comparing our results with the mucosal microbiome of humans, we found that the relative abundance of Firmicutes and Bacteroidota ranked first and second in the colon of cynomolgus monkeys (Fig. 2A) and humans (16). However, Bacteroidota was also the dominant phylum in the terminal ileum of humans, while it was scarcely detected in the ileum of cynomolgus monkeys (Fig. 2A). In contrast, the relative abundance of Bacteroidota in the ileum of rhesus macaques was comparable to that of humans and significantly different from cynomolgus monkeys (28). Thus, the decreased Bacteroidota in the ileum can be considered a unique microbial feature of cynomolgus monkeys.

Intestinal fungi are common inhabitants in the GI tract of a host, and their dysbiosis can lead to various diseases (i.e., colitis, alcoholic liver disease, and allergic lung disease) (23 – 27). Most studies relating to fungal microbes have focused on the large intestine, which has been proven to harbor the highest fungal loads in mice (25). Similarly, we found that the number of fungal OTUs and genera increased gradually from the ileum to the colon in cynomolgus monkeys (Fig. 4A and E). We also found that *Kazachstania* represented the main genus in both the small and large intestines of cynomolgus monkeys, which is in accordance with previously reported results (Fig. S4) (30). However, in human beings, *Candida* was identified as the dominated mucosa-associated genus in the large intestine, while *Kazachstania* was scarcely detected (31). Thus, the function of *Kazachstania* in cynomolgus monkeys needs further study.

As to the luminal microbes, since the biogeography of the luminal fungal communities was scarcely characterized in non-human primates or human beings, here, the luminal bacterial microbes between cynomolgus monkeys, rhesus monkeys, and human beings were compared. As shown in Fig. S3, *Prevotella* was the most abundant genus in both ileum and distal colon of rhesus monkeys, while it was specially enriched in the large intestine (caecum and colon) of cynomolgus monkeys. For human beings, *Prevotella* was enriched in the duodenum, but not the transverse colon. *Escherichia–Shigella* and *Lactobacillus* were abundant in the ileum, caecum, and colon of cynomolgus monkeys, but they were not enriched in rhesus monkeys and human beings. *Dialister*, *Coprococcus*, and *Ruminococcus* were abundant in the ileum and distal colon of rhesus monkeys, but these three genera were not enriched in either intestinal site of cynomolgus monkeys. Based on the fact that the gut microbial composition could be impacted by various factors, such as diet, season, and genetic factors, we assumed that these factors might be the key reason for the gut microbial (bacteria and fungi) differences between cynomolgus monkeys, rhesus monkeys, and human beings.

Through the analysis of bacterial composition and function, we found that the microbiota of the caecum and colon were similar to each other and significantly different from those in the ileum (Fig. 2 and 3). However, the bacterial functions related to membrane transport (ABC transporters and phosphotransferase system [PTS]) were enriched in the ileum and caecum (Fig. 3A and B). The PTS could catalyze the transportation and phosphorylation of numerous monosaccharides, disaccharides, amino sugars, polyols, and other sugar derivatives (32). In this work, we found that both the relative abundance of *Streptococcus* and the bacterial function related to PTS were enriched in the ileum and caecum, indicating that the genus *Streptococcus* might be responsible for the enriched PTS (Fig. 2E and 3B). As reported, most of the microbes belonging to the genus *Streptococcus* were sugar-fermentative bacteria and represented a significant proportion of the normal microbial population in the mouth and upper respiratory tract (33). These microbes could transport several sugars by phosphoenolpyruvate:sugar PTS via a chain of enzymatic reactions that transfer a phosphate group from phosphoenolpyruvate to the sugars. Besides, a number of PTS components, including HPr, enzyme I, and some enzyme II, have been identified and studied in various *Streptococcus* microbes, such as *Streptococcus salivarius*, *Streptococcus mutans*, and *Streptococcus sobrinus*. Thus, we assumed that the enriched bacterial PTS in the ileum and caecum was caused by the increased *Streptococcus* microbes.

Different from *Streptococcus*, our study showed that *UCG-002*, a genus belonging to family Ruminococcaceae, was specially enriched in the colon of cynomolgus monkeys (Fig. 2E). It had been reported that *UCG-002* was less abundant in the immune thrombocytopenia (ITP) patients and was significantly more prevalent in patients with major depressive disorder (34, 35), indicating that *UCG-002* was closely related to the health state of the host. Considering that the predicted bacterial functions related to the immune and nervous systems were increased in the colon site in our study, we concluded that the microbes of genus *UCG-002* might affect the health of the host by targeting the immune and nervous systems.

Although the local microbial communities along the GI tract have been studied in non-human primates (28, 29), the similarity of the microbes located at different intestinal sites has not been investigated. In our study, we investigated the percentage of bacterial and fungal genera shared by the ileum and caecum (Ile-Cae), the ileum and colon (Ile-Col), and the caecum and colon (Cae-Col), respectively (Fig. 1G and 4F). Compared to Ile-Cae and Ile-Col, the bacterial and fungal genera showed the highest similarity in Cae-Col. Of note, the genera similarity of Ile-Cae was higher than that of Ile-Col. Based on these results, we conclude that the gut bacteria and fungi located in the caecum may be viewed as the transition stage from the ileum to the colon.

In this study, we analyzed the correlation between bacterial and fungal genera along the GI tract using the Spearman correlation test. Our results showed that many fungal microbes had positive interactions with each other in the ileum and formed an obvious correlation network (Fig. 6A). Conversely, a high-magnitude positive correlation network was formed by bacterial microbes in the caecum (Fig. 6B). However, these bacterial or fungal interactions were not significant in the colon (Fig. 6C). Thus, further investigation is needed to determine whether the site-specific bacteria or fungi interactions are essential for maintaining the stability of intestinal microbiome and sustaining the host’s health. Additionally, the magnitude of the correlation network formed by bacterial and fungal microbes in the ileum and caecum was superior to that of the colon (Fig. 6).

### Limitations of this study

There are several limitations to this study. Firstly, as we worked with female monkeys, our results may not be applicable to male cynomolgus monkeys, and further investigation is needed. Secondly, we only analyzed the intestinal microbiome derived from luminal contents, and future studies should also investigate the gut biogeography of mucosal bacteria and fungi, which also play vital roles in maintaining the health of the host.

### Conclusion

Our study focused on the intestinal bacteria and fungi of female cynomolgus monkeys and provides evidence that the composition and function of bacterial and fungal communities differed significantly at different biogeographic sites of the intestine. Furthermore, by analyzing the correlation between bacterial and fungal genera using the Spearman correlation test, a specialized bacteria–fungi interaction was also observed along the GI tract. Given that the intestinal bacteria and fungi interact closely with the host, the biogeography of bacterial and fungal communities and their site-specific interactions might play vital roles in the normal physiology and health of host. In summary, our study contributes to a more comprehensive understanding of the composition and function of intestinal bacteria and fungi in NHPs and reveals their correlations along the longitudinal axis of the intestine.

## MATERIALS AND METHODS

### Animals

Cynomolgus monkeys were housed at the Songjiang Non-human Primate Facility of Institute of Neuroscience. All the monkeys were housed in an air-conditioned environment with controlled temperature (22 ± 1°C), humidity (50%±5% RH), 12 h light/12 h dark cycle (lights-on time 07:00 to 19:00), and continuous access to municipal water. All the monkeys were fed rhythmically with commercial monkey diet (Anmufei, Suzhou) twice daily (200 g per monkey at 8:00 a.m. and 15:00 p.m.) and with fruits and vegetables once daily (100 g per monkey at 10:00 a.m.) to provide essential nutrition and vitamins. All the monkeys used in this work were selected from the monkeys that died in a fight with the other monkeys and had no antibiotic exposure within 2 months.

### Fecal sample collection

The intestinal luminal contents of ileum, caecum, and colon were collected from the corresponding intestinal site of six cynomolgus monkeys, frozen with liquid nitrogen, and stored at −80°C. In total, 18 fecal samples from three gastrointestinal regions (the ileum, caecum, and colon) were collected and used for further analysis.

### Microbiome sequencing analysis

Total genomic DNA samples were extracted using the OMEGA Soil DNA Kit (M5635-02) (Omega Bio-Tek, Norcross, GA, USA). To perform the 16S rRNA gene sequencing, the hypervariable V3–V4 region of 16S rRNA was amplified using the primer pairs (labeled with sample-specific 7 bp barcodes) 338F (5′-ACTCCTACGGGAGGCAGCA-3′) and 806R (5′-GGACTACHVGGGTWTCTAAT-3′) by an ABI GeneAmp 9700 PCR thermocycler (ABI, CA, USA). As to 18S rRNA gene sequencing, the fungal ITS1–2 regions were amplified by PCR using the following primer pairs ITS1F (5′-CTTGGTCATTTAGAGGAAGTAA-3′) and ITS2R (5′-GCTGCGTTCTTCATCGATGC-3′).

The PCR product was purified using the AxyPrep DNA Gel Extraction Kit (Axygen Biosciences, Union City, CA, USA) and quantified using Quantus Fluorometer (Promega, USA). Equal amounts of amplicons were used for paired-end sequencing using the Illumina MiSeq PE300 platform (Illumina, San Diego, USA) according to the standard protocols by Majorbio Bio-Pharm Technology Co. Ltd. (Shanghai, China). The raw sequencing reads were demultiplexed, quality-filtered by fastp version 0.20.0 (36), and merged by FLASH version 1.2.7 (37).

### Bioinformatics and statistical analysis

The sequencing data were analyzed by using Uparse software (38), and the sequences with more than 97% similarity were assigned to the same OTUs. The taxonomy of each OTU representative sequence was analyzed by RDP Classifier version 2.2 (39) against the 16S rRNA database (Silva v138) using a confidence threshold of 0.7. α-Diversity was calculated using the Mothur 1.30.2; β-diversity was determined using QIIME 1.9.1. PCoA of unweighted UniFrac distances between the ileum, caecum, and colon was displayed by the WGCNA package, stat packages, and ggplot2 package in R software. The PERMANOVA test was used to assess the percentage of variation explained by the treatment along with its statistical significance using the Vegan v2.5-3 package. The Venn diagram was generated using R package “VennDiagram.” Microbial functions were predicted by PICRUSt2 upon KEGG (https://www.kegg.jp/) databases. The correlation network was performed by using Gephi software (version 0.9.4). Spearman correlation analysis was used to uncover the bacterial–fungal correlations throughout the GI tract of cynomolgus monkeys. A connection is indicated for Spearman’s correlation with a coefficient >0.6 (positive correlation) or <−0.6 (negative correlation) and a significant (*P* < 0.05) correlation. Bacteria is labeled as a yellow node; fungi is labeled as a green node. The size of each node is proportional to the relative abundance. The red lines represent positive correlations between the nodes, and the green lines represent negative correlations, with line width indicating the correlation magnitude. In this work, the statistical significance between two groups was analyzed using the Student *t*-test (unpaired, two-tailed). All statistical analyses were calculated using GraphPad (version 7.0).

## Data Availability

The 16S and 18S rRNA gene sequencing data used in this study have been deposited in the Sequence Read Archive of NCBI with accession no. SRP427150 and SRP427141.

## SUPPLEMENTAL MATERIAL

Fig. S1 to S4 spectrum.01996-23-s0001.docx Supplemental figures.

Table S1 spectrum.01996-23-s0002.xlsx Bacteria–fungi correlations in ileum.

Table S2 spectrum.01996-23-s0003.xlsx Bacteria–fungi correlations in caecum.

Table S3 spectrum.01996-23-s0004.xlsx Bacteria–fungi correlations in colon.

ASM does not own the copyrights to Supplemental Material that may be linked to, or accessed through, an article. The authors have granted ASM a non-exclusive, world-wide license to publish the Supplemental Material files. Please contact the corresponding author directly for reuse.
