# Supplementary material for: Characterizing bacterial and fungal communities along the longitudinal axis of the intestine in cynomolgus monkeys
Source: Microbiol Spectr. 2023 Nov 8;11(6):e01996-23. doi: 10.1128/spectrum.01996-23 (PMC10714780; doi:10.1128/spectrum.01996-23)

Supplementary Figure 1. Comparing bacterial differences between ileum, caecum, and colon and analyzing the site-specific bacterial correlations. (A) LEfSe analysis to characterize the taxonomic differences of microbiota between ileum, caecum, and colon. LDA score cut-off was set as 4.0 (P<0.05). Correlation heatmap analysis of top 30 bacterial genera (Spearman, P<0.05) was performed in ileum (B), caecum (C), and colon (D).


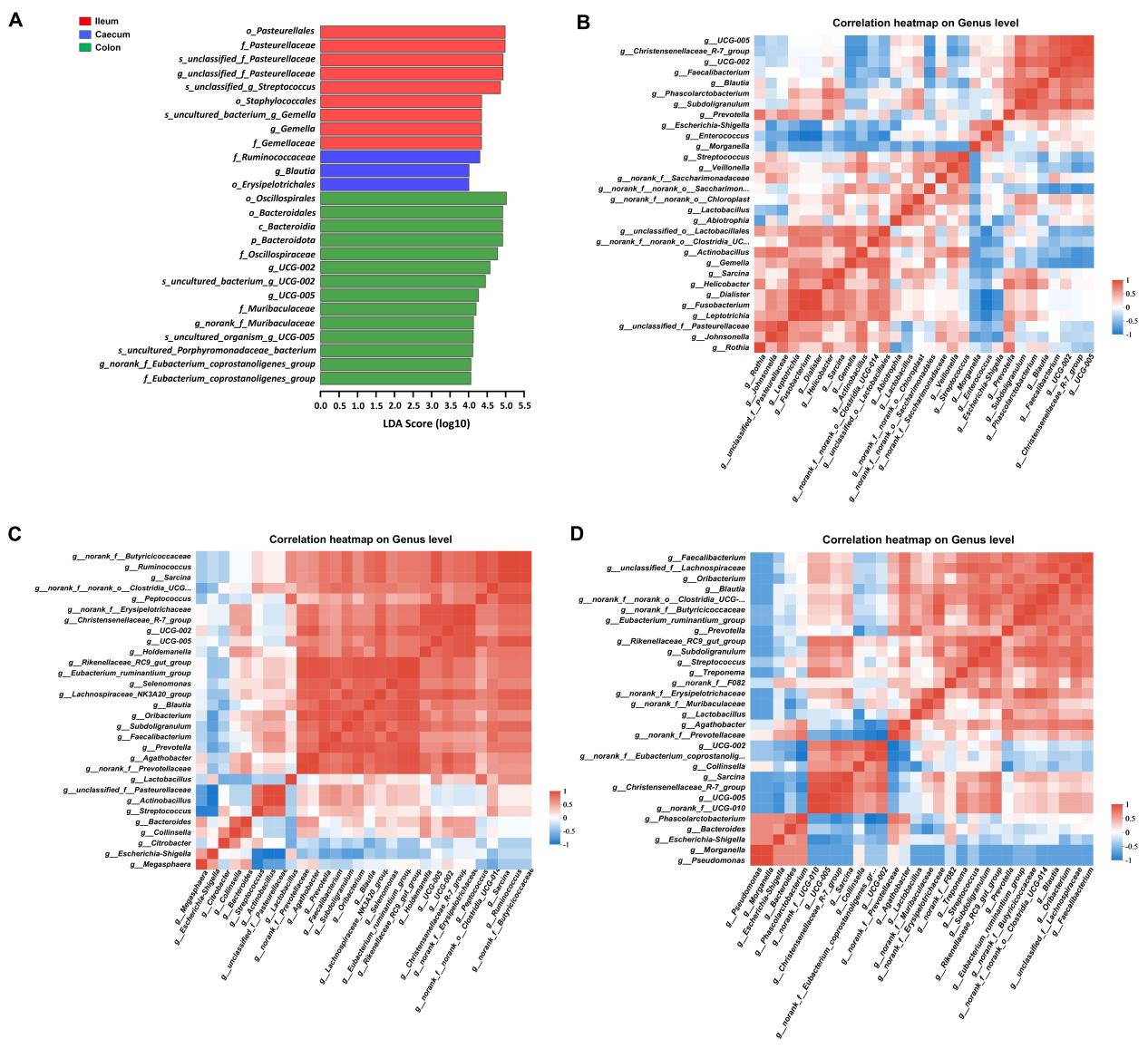


Supplementary Figure 2. Characterizing fungal correlations in ileum, caecum, and colon. Correlation heatmap analysis of top 30 fungal genera (Spearman, P<0.05) was performed in ileum (A), caecum (B), and colon (C).





Supplementary Figure 3. The main characteristics of luminal bacterial and fungal communities along the longitudinal axis of the gut of cynomolgus monkeys (A) and the comparison of luminal bacterial communities with rhesus monkeys (B) and human beings (C). The top six bacterial and fungal microbes were identified and ranked according to their relative abundance.


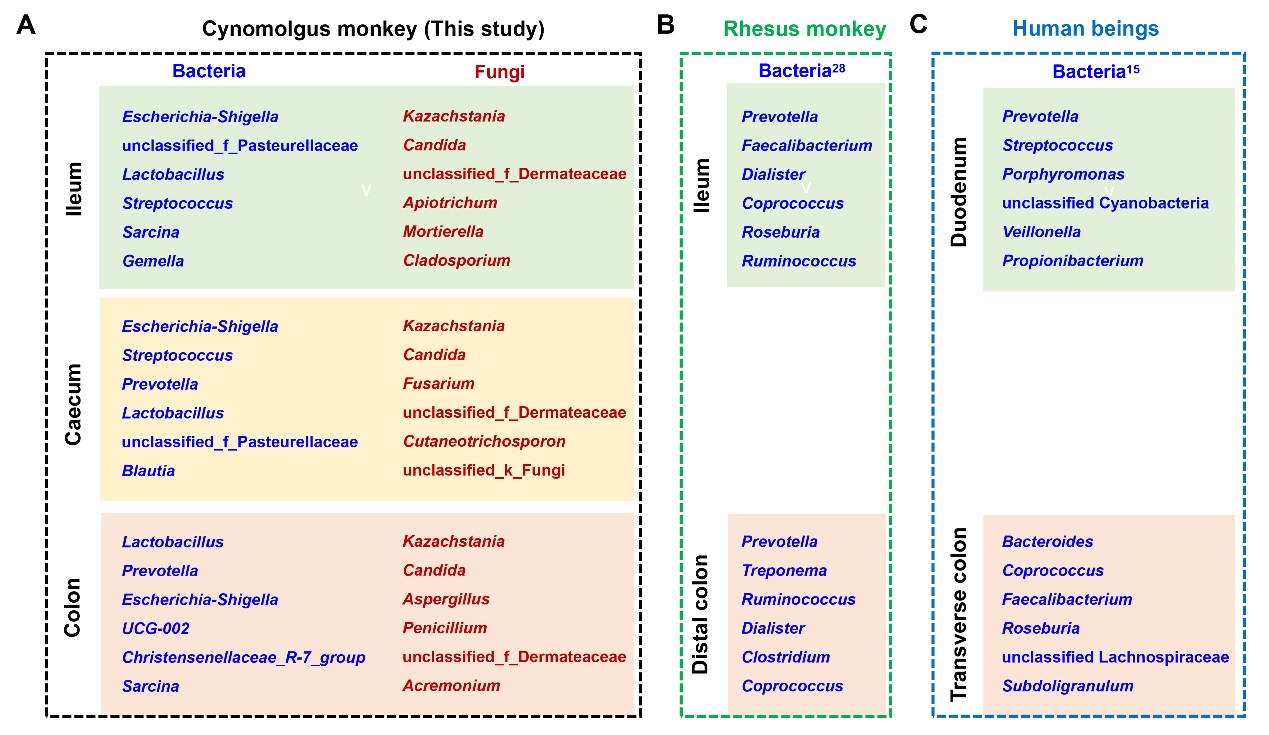


Supplementary Figure 4. Relative abundance of top 10 fungal genera at ileum (A), caecum (B), and colon (C).


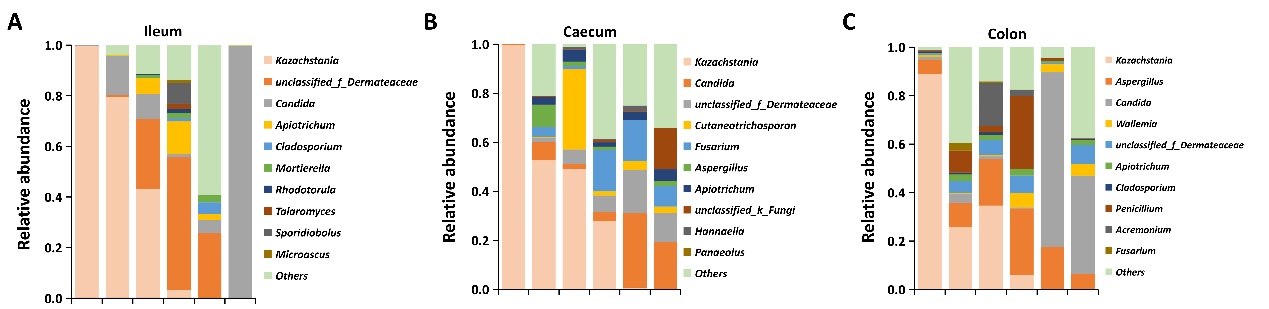

Supplement: Fig. S1 to S4 — Supplemental figures. [file spectrum.01996-23-s0001.docx]
